# Mining genomic repositories to further our knowledge of the extent of SARS-CoV-2 co-infections

**DOI:** 10.1099/mgen.0.001158

**Published:** 2024-01-16

**Authors:** Daniel Peñas-Utrilla, Patricia Muñóz, Laura Pérez-Lago, Darío García de Viedma

**Affiliations:** ^1^​ Servicio de Microbiología Clínica y Enfermedades Infecciosas, Hospital General Universitario Gregorio Marañón, Madrid, Spain; ^2^​ Instituto de Investigación Sanitaria Gregorio Marañón (IiSGM), Madrid, Spain; ^3^​ Escuela de Doctorado, Universidad de Alcalá, Plaza de San Diego, s/n, 28801 Alcalá de Henares, Madrid, Spain; ^4^​ Centro de Investigación Biomédica en Red (CIBER) de Enfermedades Respiratorias-CIBERES, 28029 Madrid, Spain; ^5^​ Departamento de Medicina, Universidad Complutense, Madrid, Spain

**Keywords:** co-infection, pipeline, repository, SARS-CoV-2

## Abstract

Recombination events between Delta and Omicron SARS-CoV-2 lineages highlight the need for co-infection research. Existing studies focus on late-phase co-infections, with few examining earlier pandemic stages. This new study aims to globally identify and characterize co-infections using a bioinformatic pipeline to analyse genomic data from diverse locations and pandemic phases. Among 26988 high-quality SARS-CoV-2 isolates from 11 diverse project databases, we identified 141 potential co-infection cases (0.52%), surpassing previous prevalence estimates. These co-infections were observed throughout the pandemic timeline, with an increase noted after the emergence of the Omicron variant. Co-infections involving the Omicron variant were the most prevalent, potentially influenced by the high level of diversity within this lineage and its impact on the viral landscape. Additionally, we found co-infections involving the pre-Alpha/Alpha lineages, which have been rarely described, raising possibilities of contributing to new lineage emergence through recombination events. The analysis revealed co-infection cases involving both different and the same lineages/sublineages. Our study showcases the potential of our pipeline to leverage valuable information stored in global sequence repositories, advancing our understanding of SARS-CoV-2 co-infections. The prevalence of co-infections highlights the importance of monitoring viral diversity and its potential implications on disease dynamics. Integrating clinical data with genomic findings can further shed light on the clinical implications and outcomes of co-infections.

## Abbreviations

ENA, European Nucleotide Archive; HZ, Heterozygous; MHP, Mean proportion of major alleles; NCBI, National Center for Biotechnology Information; SHP, Standard deviation of the MHP; SNP, Single Nucleotide Polymorphism; USA, United States of America.

## Impact Statement

This study sheds light on the prevalence and characteristics of SARS-CoV-2 co-infections during different phases of the COVID-19 pandemic. By applying our specialized bioinformatics pipeline, this research exploits the potential of genomic global repositories to extend our knowledge of the complex landscape of SARS-CoV-2 co-infections. In contrast to previous studies that focused on specific lineages in certain populations during limited periods, our analysis covers a wide range of lineages, locations and time periods. The identification of 141 potential co-infection cases from a dataset of 26988 isolates surpassed previous estimates of co-infection prevalence. Our analytical pipeline also provides the segregation of the two co-infecting strains, something unprecedented in the literature on this topic, allowing for the precise description of the lineages involved. From our data, it can be deduced that co-infection occurred especially after the Omicron emergence; however, it could also be found in preceding stages, involving not only strains from different lineages but also from the same one. Altogether, our findings suggest that recombination due to co-infection might have been a more common driver of SARS-CoV-2 diversification along the pandemic than previously expected.

## Data Summary

This study was carried out with data retrieved from the ENA Database (Table 1). Diagnosis times for the selected sequences were obtained from the NCBI database.

**Table 1.** SARS-CoV-2 co-infection cases identified from ENA repositories and general features of the projects selected for analysis

Project name	Total sequences	Country	Pandemic window	Selected sequences/good quality	Co-infection candidates (%)
PRJEB37886	2484540	UK	2020-Feb to 2023-Mar	6243/583	2 (0.34)
PRJEB43828	68398	Switzerland	2020-Mar to 2021-May	4200/3543	17 (0.48)
PRJEB44396	18285	Spain	2020-Apr to 2022-Nov	2605/2595	27 (1.04)
PRJEB47340	44496	Portugal	2020-Mar to 2023-Mar	4137/3987	8 (0.2)
PRJEB52934	9851	Estonia	2021-Jan to 2022-Apr	2795/654	1 (0.15)
PRJNA613958	14860	Australia – Victoria	2020-Mar to 2020-Oct	2369/2093	0 (0)
PRJNA614995	7079	USA – Utah	2020-Mar to 2021-May	3321/1768	4 (0.23)
PRJNA636748	12098	South Africa	2020-Jan to 2023-Mar	3289/1720	35 (2.03)
PRJNA645906	16095	USA – San Francisco	2020-Mar to 2021-May	4549/3794	10 (0.26)
PRJNA704697	112484	USA – New York	2020-Sep to 2023-Apr	4318/3732	25 (0.67)
PRJNA713804	17494	USA – Baltimore	2020-Dec to 2023-Mar	2874/2519	12 (0.48)

## Introduction

The identification of recombination events between the Delta and Omicron (BA.1 and BA.2) lineages justifies the investigation of SARS-CoV-2 co-infections. However, most existing studies are restricted to co-infections involving these two divergent lineages and to late phases of the pandemic when they co-circulated [1–4]. Only a few studies have analysed co-infections at earlier stages of the pandemic [5–7].

Previous research has estimated the prevalence of SARS-CoV-2 co-infections involving the Delta and Omicron lineages at approximately 0.2 % [8]. In the population covered by our hospital in Madrid, Spain, our group found comparable co-infection frequencies throughout the entire pandemic. For that study [9], we applied a pipeline that we had recently designed, which enabled us not only to identify co-infection regardless of the strains/lineages involved (>7 SNP differences between them), but also to segregate the sequences involved for individual analysis.

The objective of this new study, applying the same pipeline as a tool, was to exploit sequences deposited in genomic repositories in order to make a global identification and characterization of co-infections in different geographical settings and at different phases of the COVID-19 pandemic.

## Methods

### Data acquisition

Paired-end FASTQ files were downloaded using fastq-dump software (version 3.0.5, https://github.com/ncbi/sra-tools) from 11 different projects of the ENA database (Table 1).

### Bioinformatic analysis and detection of co-infection candidates

FASTQ files were subjected to bioinformatic analysis and detection of co-infection candidates as previously described [9]. Briefly, adapters and low-quality regions were processed from paired reads using fastp (version 0.20.1, https://github.com/OpenGene/fastp). Quality control was assessed with fastQC (version v0.11.9, https://github.com/s-andrews/FastQC). Good quality reads were mapped with BWA (version 0.7.17-r1188, https://github.com/lh3/bwa) to the Wuhan-1 SARS-CoV-2 reference sequence (GenBank accession no. NC_045512.2); IVar (version 1.3.1, https://github.com/andersen-lab/ivar) was used to call variants, and pangolin (version 4.1.2, https://github.com/cov-lineages/pangolin) was used for lineage annotation.

The co-infection detection pipeline (https://github.com/MG-IiSGM/SARS_COVinfections) focuses on the single nucleotide polymorphisms (SNPs) identified, using IVar from sequences with ≥90 % genome coverage and a depth of >30X. For the SNPs with heterozygous (HZ) calls (two alleles co-existed, with one of the alternative alleles at a frequency between 15 and 85 %), it calculates the mean proportion of major alleles (MHP), the standard deviation of the MHP (SHP), and the percentage of SNPs with a heterozygous proportion of the major allele found within the MHP ± (SHP +1.5 %) to assure a homogeneous distribution of frequencies in the SNPs with HZ calls. Co-infection candidates are chosen based on the number of SNPs with heterozygous calls (>7 positions), the MHP (< 75 %), the mean SHP (≤ 0.08 %) and the percentage (≥70 %) of those SNPs with the major allele found within that MHP ± (SHP +1.5 %). Our approach requires a minimum average proportion of the minor strain in a co-infection and checks for a consistent allele relative frequency across the positions with HZ calls.

## Results and discussion

We selected 11 different SARS-CoV-2 projects from the ENA database (Table 1). Taking into account the frequencies of SARS-CoV-2 co-infection in the literature, we ruled out projects with fewer than 5000 sequences obtained with Illumina. In our selection, we considered different time windows for the collection dates of the sequenced specimens to ensure comprehensive coverage over the entire timeline of the pandemic and different geographical settings. Our selection included five projects from Europe: Portugal (March 2020 to March 2023), Spain (April 2020 to November 2022), Switzerland (March 2020 to May 2021), Estonia (January 2021 to April 2022), and the UK (February 2020 to March 2023); four from the USA: Baltimore (December 2020 to March 2023), New York (September 2020 to April 2023), San Francisco (March 2020 to May 2021), and Utah (March 2020 to May 2021); one from Africa: South Africa (January 2020 to March 2023); and one from Australia: Victoria (March 2020 to October 2020).

To ensure that the analysis was computationally feasible and an equitable representation of sequences from each month throughout the pandemic timeline (from March 2020 to March 2023), we randomly selected 1100 sequences per month among all the sequences for that month from all projects’ sequences (Table 1). In total, 40700 sequences were downloaded, with each project contributing approximately 9% of the total sequences downloaded; 26988 sequences (66 %) met the quality threshold, with genome coverage ≥90% and >30X depth. The proportion of high-quality sequences was not consistent across all projects. Some projects contained a higher number of low-quality deposited sequences, which limits the sample size to be used in our analysis. Notably, in the project from the UK, a mere 9.34% of the sequences met the predefined quality criteria. This inconsistency can be attributed primarily to the lack of stringent quality standards generally governing the submission of sequences within the ENA database. These high-quality sequences were processed using our pipeline [9].

**Fig. 1.**
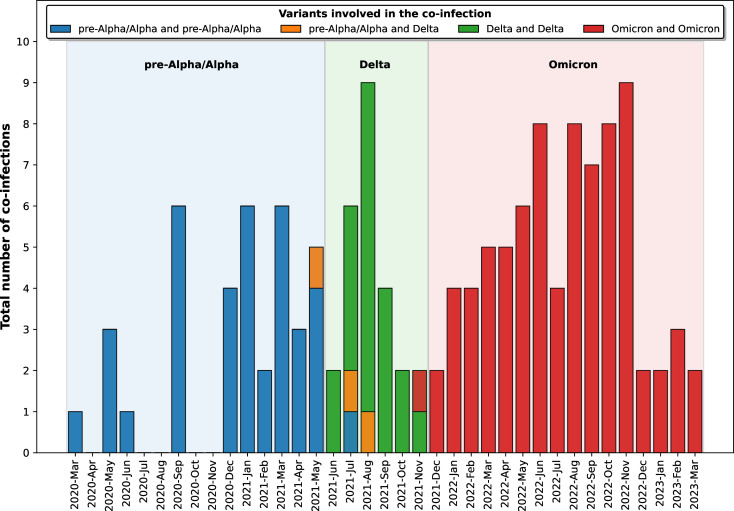
Distribution of the co-infections identified throughout the pandemic period, specifying the lineages involved. The dominant circulating lineage for each period is indicated in the upper section.

Overall, 141 potential co-infection cases (0.52 %) were identified (Table 2) across various countries (Table 1) and for most of the months during the pandemic (Fig. 1). Notably, no co-infections were detected in the Australian project. It is important to note that the sequences from this project mainly corresponded to the early stages in the pandemic (March 2020 to October 2020), when low diversity is still expected among the circulating variants, which makes the identification of co-infections difficult. In addition, we must remind that due to the random subsampling of the sequences, we cannot fully rule out co-infections in some of the periods or projects in which we did not identify them.

**Table 2.** Detailed analysis of the positions with heterozygous calls from the sequences corresponding to SARS-CoV-2 co-infections. Asterisks (*) indicate lower-confident segregations (mean heterozygous allele frequency ≤ 60%). HZ: heterozygous, STD: standard deviation

Study Accession	Run Accession	Collection date	No. HZ* calls	Mean HZ* proportion	STD† HZ proportion	% SNPs within STD	Minority lineage	Majority lineage
PRJNA614995	SRR11607614	2020-03-16	8	0,65	0,07	75	B.1	B.1
PRJNA645906	SRR12661209	2020-05-13	27	0,74	0,08	81	B.1.1.142	B.1.1
PRJNA614995	SRR12544252	2020-05-20	10	0,66	0,06	80	B.1.426	B.1.426
PRJNA645906	SRR12661141	2020-05-21	11	0,74	0,08	82	B.1	B.1
PRJNA636748	SRR12151800	2020-06-11	18	0,74	0,07	83	B.1.1.117	B.1.1.117
PRJNA645906	SRR12833799	2020-09-01	8	0,68	0,07	88	B.1.503	B.1.503
PRJEB43828	ERR8517868	2020-09-09	9	0,62	0,06	78	B.1.1	B.1
PRJEB43828	ERR8517825	2020-09-17	8	0,66	0,07	88	B.1	B.1.1.39
PRJEB43828	ERR8517824	2020-09-18	12	0,67	0,05	92	B.1.1.39	B.1.1
PRJNA645906	SRR13094088	2020-09-24	31	0,72	0,08	77	B.1	B.1
PRJNA704697	SRR16870822	2020-09-28	23	0,74	0,08	91	B.1.1.432	B.1.1.432
PRJNA645906	SRR13690340	2020-12-09	33	0,7	0,05	88	B.1.2	B.1.232
PRJNA636748	SRR13632517	2020-12-15	15	0,53*	0,03	93	B.1.351	B.1.351
PRJNA636748	SRR13632491	2020-12-16	12	0,72	0,07	75	B.1	B.1.351
PRJNA636748	SRR14119595	2020-12-21	17	0,62	0,08	71	B.1.351	B.1.351
PRJNA636748	SRR13997363	2021-01-05	8	0,68	0,08	88	B.1.351	B.1.351
PRJNA645906	SRR14201739	2021-01-16	43	0,56*	0,06	91	B.1.1.519	B.1
PRJNA645906	SRR14201758	2021-01-16	45	0,6*	0,05	82	B.1.1.7	B.1.2
PRJNA645906	SRR14201740	2021-01-16	47	0,59*	0,07	85	B.1.111	B.1.280
PRJNA614995	SRR13891583	2021-01-24	15	0,71	0,05	80	B.1.2	B.1.2
PRJNA614995	SRR13891590	2021-01-24	41	0,64	0,06	90	B.1.2	B.1.400
PRJNA645906	SRR13956290	2021-02-03	17	0,63	0,07	71	B.1.2	B.1.2
PRJEB52934	ERR10108652	2021-02-16	48	0,58*	0,07	85	B.1.160	B.1.258
PRJNA636748	SRR14590508	2021-03-07	31	0,74	0,08	74	B.1.633	B.1.351
PRJNA636748	SRR14590522	2021-03-12	32	0,73	0,08	72	B.1.633	B.1.351
PRJNA704697	SRR15612541	2021-03-12	43	0,69	0,08	74	B.1.637.1	B.1
PRJNA636748	SRR14590516	2021-03-13	38	0,73	0,08	79	B.1.633	B.1.351
PRJNA636748	SRR14590513	2021-03-15	36	0,73	0,08	78	B.1.633	B.1.351
PRJNA636748	SRR14590493	2021-03-18	32	0,74	0,08	78	B.1.633	B.1.351
PRJNA704697	SRR15623054	2021-04-12	10	0,62	0,08	80	B.1.526	B.1.526
PRJEB43828	ERR10739689	2021-04-28	15	0,58*	0,08	80	B.1.1.7	B.1.1.7
PRJNA704697	SRR16861049	2021-04-30	16	0,73	0,08	81	B.1.526	B.1.526
PRJNA645906	SRR14791620	2021-05-06	8	0,74	0,07	75	B.1.427	B.1.427
PRJNA636748	SRR15032901	2021-05-18	8	0,73	0,08	75	B.1.351	B.1.351
PRJNA636748	SRR15001125	2021-05-18	56	0,59*	0,07	89	AY.122	B.1
PRJNA636748	SRR15032872	2021-05-24	33	0,65	0,08	70	B.1.351	B.1.351
PRJNA636748	SRR15032862	2021-05-25	13	0,73	0,08	85	B.1.351	B.1.351
PRJNA636748	SRR15060984	2021-06-22	11	0,7	0,08	73	AY.45	AY.45
PRJEB37886	ERR6206729	2021-06-24	15	0,61	0,08	73	AY.87	AY.4
PRJNA636748	SRR15566771	2021-07-04	12	0,74	0,08	83	AY.45	AY.45
PRJEB43828	ERR10013584	2021-07-06	17	0,59*	0,06	88	AY.42	AY.98
PRJEB43828	ERR10769221	2021-07-12	8	0,64	0,07	75	C.37.1	C.37.1
PRJEB43828	ERR10772247	2021-07-14	8	0,63	0,08	75	AY.42	AY.42
PRJNA636748	SRR16009040	2021-07-19	71	0,72	0,07	77	C.1.2	AY.45
PRJNA636748	SRR15702314	2021-07-31	31	0,74	0,08	77	B.1.617.2	AY.45
PRJEB43828	ERR9488508	2021-08-03	8	0,65	0,08	75	AY.122	AY.122
PRJNA636748	SRR15676668	2021-08-05	68	0,65	0,07	79	B.1.617.2	C.1.2
PRJEB43828	ERR10745837	2021-08-09	11	0,6*	0,08	73	AY.43	B.1.617.2
PRJNA704697	SRR16882512	2021-08-09	13	0,74	0,08	85	AY.26	AY.26
PRJEB43828	ERR10676993	2021-08-12	13	0,74	0,07	77	AY.122	AY.121
PRJEB43828	ERR10673853	2021-08-12	18	0,64	0,05	89	AY.98.1	AY.4
PRJEB43828	ERR10783316	2021-08-12	28	0,65	0,08	86	AY.122	AY.4
PRJEB43828	ERR10788231	2021-08-16	19	0,63	0,08	79	AY.6	AY.112
PRJNA636748	SRR19326378	2021-08-26	13	0,71	0,08	85	AY.91	AY.120.2
PRJNA636748	SRR16298316	2021-09-06	9	0,65	0,08	78	AY.45	AY.45
PRJEB37886	ERR6883101	2021-09-08	19	0,57*	0,08	89	AY.4	AY.122
PRJNA636748	SRR16298646	2021-09-12	8	0,7	0,08	75	AY.45	AY.45
PRJEB47340	ERR6912151	2021-09-21	8	0,74	0,07	88	AY.94	AY.94
PRJNA636748	SRR17090374	2021-10-23	9	0,59*	0,08	89	AY.45	AY.45
PRJEB47340	ERR7252304	2021-10-26	16	0,62	0,07	75	AY.124	AY.124
PRJEB47340	ERR7451625	2021-11-27	8	0,72	0,08	75	BA.1	BA.1
PRJEB43828	ERR9434426	2021-11-29	8	0,66	0,07	88	AY.46.6	AY.46.6
PRJNA713804	SRR17885105	2021-12-13	11	0,74	0,08	73	BA.1.1	BA.1
PRJNA713804	SRR18013415	2021-12-29	10	0,66	0,05	80	BA.1.1	BA.1.1
PRJNA704697	SRR17917030	2022-01-02	14	0,74	0,08	71	BA.1.1	BA.1.1
PRJNA704697	SRR17922769	2022-01-12	10	0,71	0,07	80	BA.1	BA.1.1
PRJEB44396	ERR10307837	2022-01-25	8	0,64	0,08	88	BA.1.17	BA.1.17
PRJEB44396	ERR10307835	2022-01-25	10	0,74	0,08	80	BA.1	BA.1
PRJNA636748	SRR18240325	2022-02-07	10	0,72	0,06	80	BA.1.1	BA.1.1
PRJEB44396	ERR10317580	2022-02-08	23	0,71	0,08	74	B.1.1.529	BA.1.17
PRJEB44396	ERR10317595	2022-02-10	8	0,74	0,05	75	BA.2	BA.2
PRJEB47340	ERR9247894	2022-02-28	11	0,65	0,04	82	BA.2.5	BA.2
PRJEB44396	ERR10321941	2022-03-04	8	0,74	0,06	75	BA.2	BA.2
PRJNA713804	SRR18672980	2022-03-07	10	0,73	0,08	70	BA.1.1	BA.1.1
PRJEB44396	ERR10322853	2022-03-18	8	0,74	0,08	88	BA.2	BA.2
PRJEB44396	ERR10372223	2022-03-20	20	0,62	0,08	75	BA.1.17	BA.1.17
PRJEB44396	ERR10373275	2022-03-21	23	0,74	0,08	78	BA.2	BA.2
PRJEB44396	ERR10373189	2022-04-01	9	0,73	0,08	89	BA.2	BA.2
PRJEB44396	ERR10384662	2022-04-04	8	0,66	0,08	75	BA.2	BA.2.10
PRJEB43828	ERR9941650	2022-04-13	8	0,74	0,07	75	BA.2	BA.2
PRJEB44396	ERR10384852	2022-04-23	8	0,71	0,08	75	BA.2	BA.2
PRJEB44396	ERR10387212	2022-04-26	8	0,73	0,08	88	BA.2.9	BA.2.9
PRJEB44396	ERR10387062	2022-05-12	8	0,7	0,07	75	BA.2	BA.2
PRJEB44396	ERR10395158	2022-05-12	10	0,62	0,08	80	BA.2.9	BA.2
PRJNA636748	SRR20015497	2022-05-26	11	0,72	0,07	73	BE.1	BE.1
PRJNA704697	SRR19864141	2022-05-28	24	0,56*	0,07	92	BA.2.12.1	BA.4.6
PRJEB44396	ERR10421702	2022-05-30	8	0,74	0,07	88	BA.2	BA.2
PRJNA636748	SRR20770734	2022-05-30	10	0,73	0,07	70	BA.2	BA.4
PRJNA636748	SRR20770564	2022-06-09	9	0,71	0,08	78	BA.4	BA.4
PRJNA636748	SRR20770412	2022-06-11	19	0,59*	0,07	79	AY.45	AY.45
PRJEB47340	ERR9884111	2022-06-12	11	0,72	0,07	82	BA.5.1.22	BA.5.1
PRJNA704697	SRR20295773	2022-06-12	13	0,62	0,07	77	BA.2.12.1	BA.2.12.1
PRJNA704697	SRR20295237	2022-06-14	13	0,63	0,06	77	BA.2.12.1	BA.2.12.1
PRJEB44396	ERR10421965	2022-06-15	8	0,74	0,08	75	BA.2	BA.2
PRJNA704697	SRR20296744	2022-06-18	23	0,72	0,06	87	BA.2.12.1	BA.2.9
PRJEB44396	ERR10423923	2022-06-20	8	0,74	0,07	75	BA.5.1.22	BA.5.1.22
PRJNA704697	SRR20968897	2022-07-07	9	0,71	0,08	78	BA.5.1	BA.5.1
PRJEB47340	ERR9992297	2022-07-11	13	0,67	0,03	85	BA.5.1.5	BA.5.2.1
PRJEB44396	ERR10433285	2022-07-29	8	0,7	0,08	75	BA.5.2.1	BA.5.2.1
PRJEB44396	ERR10433228	2022-07-29	9	0,74	0,04	78	BA.5.1	BA.5.1
PRJEB47340	ERR10086535	2022-08-02	9	0,63	0,08	89	BA.1.15	BA.1.15
PRJNA704697	SRR21162692	2022-08-05	18	0,56*	0,05	78	BA.5	BE.1
PRJNA636748	SRR21373998	2022-08-07	41	0,7	0,08	80	BA.5.2	BA.1
PRJEB44396	ERR10627519	2022-08-10	9	0,74	0,08	78	BA.5.1	BA.5.1
PRJNA636748	SRR21373980	2022-08-10	22	0,74	0,08	77	BE.1.1	BA.5.2
PRJNA704697	SRR21572605	2022-08-14	11	0,61	0,05	82	BA.5	BA.5.1.1
PRJNA713804	SRR21770881	2022-08-14	27	0,72	0,07	81	BF.26	BA.2.12.1
PRJNA704697	SRR21574721	2022-08-24	17	0,7	0,06	88	BA.5.2.1	BF.13
PRJEB44396	ERR10659367	2022-09-09	8	0,74	0,07	75	BA.5.1.23	BA.5.1.23
PRJNA704697	SRR22005075	2022-09-19	11	0,72	0,08	73	BA.5.2.6	BA.5.2.1
PRJNA704697	SRR22005495	2022-09-19	21	0,71	0,05	76	BA.5.2.34	BA.5.1.1
PRJNA704697	SRR22005289	2022-09-19	24	0,71	0,08	75	BA.5.2	BA.4.6
PRJEB44396	ERR10659347	2022-09-21	9	0,74	0,08	78	BA.5.3.3	BA.5.3.3
PRJEB44396	ERR10659350	2022-09-21	9	0,73	0,08	78	BA.5.2	BA.5.2
PRJNA713804	SRR21891288	2022-09-27	8	0,68	0,06	88	BA.5.1	BA.5.1
PRJEB44396	ERR10662000	2022-10-03	18	0,65	0,06	78	BA.5.1.30	BA.5.2
PRJNA636748	SRR22243713	2022-10-15	10	0,73	0,08	70	BA.5.3.1	BA.5.3.1
PRJNA636748	SRR22243807	2022-10-17	8	0,71	0,07	75	BA.5.11	BA.5.11
PRJNA704697	SRR22216662	2022-10-21	31	0,67	0,08	74	BA.4.6	BA.5
PRJNA704697	SRR22303195	2022-10-24	12	0,7	0,07	92	BE.4.2	BE.4
PRJNA704697	SRR22303303	2022-10-26	8	0,61	0,08	88	XBV	XBV
PRJNA704697	SRR22431400	2022-10-27	10	0,65	0,07	70	BA.2.75.2	BA.2.75.2
PRJEB43828	ERR10849187	2022-10-28	32	0,56*	0,07	91	BA.5.2	BQ.1
PRJNA713804	SRR22483907	2022-11-06	29	0,64	0,07	79	BE.1.1.2	BA.5.2.31
PRJNA713804	SRR22483910	2022-11-07	28	0,62	0,08	86	BE.1	BF.7
PRJEB44396	ERR10669233	2022-11-08	8	0,7	0,08	75	BQ.1.8	BQ.1.8
PRJNA704697	SRR22684072	2022-11-15	28	0,74	0,07	75	BQ.1.1.68	BA.5.1.18
PRJNA713804	SRR22730955	2022-11-16	8	0,74	0,08	88	BQ.1.8	BQ.1.8
PRJEB44396	ERR10669525	2022-11-16	49	0,7	0,05	88	BQ.1.1.72	XBB
PRJEB44396	ERR10774645	2022-11-23	10	0,73	0,08	80	BQ.1.1	BQ.1.1
PRJNA713804	SRR22733380	2022-11-25	47	0,73	0,07	70	BF.7.23	XBB.1.30
PRJNA704697	SRR22813698	2022-11-30	21	0,58*	0,05	90	BA.5.2	BQ.1
PRJNA704697	SRR22913345	2022-12-03	30	0,74	0,07	83	BA.5.1.30	BF.14
PRJEB47340	ERR10760517	2022-12-15	8	0,55*	0,06	88	BA.2.5	BA.2.5
PRJNA713804	SRR23291085	2023-01-03	28	0,68	0,07	75	XBV	XBB.1.5
PRJEB43828	ERR10851483	2023-01-06	9	0,7	0,08	78	BQ.1.26	BQ.1.26
PRJNA713804	SRR23874498	2023-02-20	8	0,74	0,07	75	XBB.1	XBB.1.5
PRJNA636748	SRR24124565	2023-02-21	13	0,7	0,07	77	BE.7	BE.7
PRJNA713804	SRR23874642	2023-02-24	8	0,69	0,08	75	XBB.1	XBB.1.5
PRJNA636748	SRR24124613	2023-03-13	8	0,64	0,08	75	XBB.1.5	XBB.1.5
PRJNA636748	SRR24124670	2023-03-16	12	0,68	0,08	75	XBB.1.5	XBB.1.5

The co-infection frequency was higher than reported elsewhere [8, 9], although the frequencies of individual projects ranged widely (0.15–2.03) (Table 1). Moreover, higher co-infection rates were found in the Spanish and South African projects (1.04 and 2.03 %, respectively). These higher rates could initially be attributed to the longer time covered by these projects, encompassing sequences collected throughout almost the entire pandemic timeline. Thus, it would be conceivable that the elevated rates of co-infections within these projects might result from the higher opportunity to detect them when the sampling periods are longer. Nevertheless, in other projects with equivalent time coverages, co-infection percentages were lower (ranging from 0.15–0.67 %,) suggesting a role for other factors, out of time-coverage, behind differences in co-infection proportions.

As our pipeline allows for segregation of the sequences involved in each co-infection, we first determined the lineages involved in each case. Segregation of the two strains involved in a co-infection was confidently performed in 124 (88 %) of the co-infection cases where mean heterozygosity was between 60 and 75 % (Table 2). Fifty-five percent of the co-infections involved sequences from the same lineage/sublineage, while the remaining cases involved different lineages/sublineages (Table 2). In the first group, we identified 20, 11 and 46 co-infections involving the same pre-Alpha/Alpha, Delta and Omicron lineages, respectively. In the second group, we observed 17 co-infections involving two distinct pre-Alpha or Alpha sublineages, three involving pre-Alpha/Alpha and Delta, ten involving two distinct Delta lineages/sublineages and 34 involving two different Omicron lineages/sublineages (Table 2). Most of the co-infections involved Omicron variants (57 %, 80/141 sequences). Co-infections involving Delta and Omicron lineages were not detected in our analysis. However, the absence of their detection in this study does not necessarily imply that they did not occur or they cannot be detected, as we have previously identified such cases with this pipeline in our prior work [9].

The total number of HZ calls observed in the co-infections identified ranged from 8 to 71. As expected, cases with a higher number of HZ calls (>17) corresponded to co-infections involving different lineages/sublineages (41 of 48). Conversely, those with a lower number of HZ calls (8-17) mainly corresponded to co-infections within the same lineage or sublineage.

In our previous study performed in Madrid [9], we identified a proportion of co-infection candidates that were likely due to co-existence of variants emerging in immunosuppressed patients or to laboratory contaminations. However, in this current study, the unavailability of comprehensive epidemiological, laboratory and clinical data and the constraints of sample resequencing hinder the replication of some of the validation procedures applied in our prior work. Nonetheless, we evaluated the consistency between the lineages involved in each of the co-infection candidates and the corresponding circulating lineages at the moment of each diagnosis. In all cases, the linages involved in the co-infections corresponded to the expected circulating variants (Fig. 1). Furthermore, the detection of low-frequency recombined reads (alleles from each of the co-infecting variants lying in the same single short read) would demonstrate the simultaneous presence of both strains and their concurrent viral replication, ruling out a mere co-existence of sequences due to laboratory cross-contamination. To perform this analysis, we first identified the cases in which we could find some of the differential SNPs for the co-infecting strains located 150-pb apart (length for Illumina short-reads). Among the 141 co-infection cases, we identified these tandem close SNPs in 59 of them, which could allow us to detect potential recombination in the same short read. Recombined low-frequency (average frequency 3.6%) single reads were observed in 43 of these (73 %). These findings indicate that recombination was found in a high number of the co-infection cases, in which the distribution of SNPs for the co-infecting strain allows this analysis to be performed. Whenever recombination is detected, cross-contamination can be fully ruled out.

We must remind that the replication of the robust validation procedures applied in our previous study to distinguish co-infections from potential cross-contaminations is unattainable in the current study. However, the epidemiological consistency and the detection of low-frequency recombined reads can potentially rule out the possible significant inflation of estimated rates, reinforcing the validity of the co-infection rates obtained in this study.

While our study primarily focused on identification and segregation for analysis of SARS-CoV-2 co-infections, it would also be useful to consider the clinical implications. Analysing clinical data in conjunction with genomic data could offer deeper insights into the impact of co-infections on disease severity, treatment response and patient outcomes [10, 11]. Future studies should integrate clinical metadata for a comprehensive understanding of co-infections.

## Conclusions

Our study investigated the presence and genomic characteristics of SARS-CoV-2 co-infections during different phases of the COVID-19 pandemic and in different countries. By digging in a dataset of 26988 good-quality SARS-CoV-2 isolates from 11 diverse project databases, we identified 141 potential co-infection cases (0.52%), surpassing previous estimates of prevalence. These co-infections were observed throughout the pandemic timeline, and increased after the Omicron variant emerged. Co-infections involving the Omicron variant were the most prevalent, consistent with the likely reduction in cross-protection associated with the high level of diversity within this lineage and its potential impact on the viral landscape. In addition, we identified co-infections involving the pre-Alpha/Alpha lineages, which have been only sporadically described and may also have contributed to the emergence of new lineages through recombination events. Our analysis revealed co-infection cases involving not only different lineages/sublineages but also strains from the same lineage. This study demonstrates the potential of our pipeline to readily exploit the valuable information stored in global sequence repositories and advances our understanding of SARS-CoV-2 co-infections. Integrating clinical data with genomic findings could provide valuable information on the clinical implications and outcomes of co-infections.
